# Impact of Plant Growth Promoting Rhizobacteria in the Orchestration of *Lycopersicon esculentum* Mill. Resistance to Plant Parasitic Nematodes: A Metabolomic Approach to Evaluate Defense Responses Under Field Conditions

**DOI:** 10.3390/biom9110676

**Published:** 2019-10-31

**Authors:** Kanika Khanna, Anket Sharma, Puja Ohri, Renu Bhardwaj, Elsayed F. Abd_Allah, Abeer Hashem, Parvaiz Ahmad

**Affiliations:** 1 Department of Botanical and Environmental Sciences, Guru Nanak Dev University, Amritsar 143005, India; kanika.27590@gmail.com (K.K.); anketsharma@gmail.com (A.S.); 2 State Key Laboratory of Subtropical Silviculture, Zhejiang A&F University, Hangzhou 311300, China; 3 Department of Zoology, Guru Nanak Dev University, Amritsar 143005, India; 4 Department of Plant Production, Faculty of Food & Agricultural Sciences, King Saud University, Riyadh 11451, Saudi Arabia; eabdallah@ksu.edu.sa; 5 Botany and Microbiology Department, College of Science, King Saud University, P.O. Box. 2460, Riyadh 11451, Saudi Arabia; habeer@ksu.edu.sa; 6 Mycology and Plant Disease Survey Department, Plant Pathology Research Institute, ARC, Giza 12511, Egypt; 7 Department of Botany, S.P. College, Srinagar, Jammu and Kashmir 190001, India

**Keywords:** root knot nematodes, biocontrol agents, *Lycopersicon esculentum*, photosynthetic parameters, oxidative damage, antioxidants, phenolics, osmoprotectants and organic acid profiling

## Abstract

The present study deals with biological control of *Meloidogyne incognita* in 45-days old *Lycopersicon esculentum*, inoculated with *Pseudomonas aeruginosa*(M1) and *Burkholderia gladioli* (M2). The improved plant growth and biomass of nematode infested Plant growth promoting rhizobacteria (PGPR) inoculated plants was observed. Remarkable reduction in the numbers of second stage juvenile (J2s), root galls was recorded after treatment of microbes relative to experimental controls. Moreover, the lowered activities of oxidative stress markers (H_2_O_2_ (hydrogen peroxide), O_2_^−^ (superoxide anion), malondialdehyde (MDA)) was estimated in plants after rhizobacterial supplementation. Higher activities of enzymatic (SOD (Superoxide dismutase), POD (Guaiacol peroxidase), CAT (Catalase), GPOX (Glutathione peroxidase), APOX (Ascorbate peroxidase), GST (Glutathione-S-transferase), GR (Glutathione reductase), DHAR (Dehydroascorbate reductase), PPO (Polyphenol oxidase)) and non-enzymatic (glutathione, ascorbic acid, tocopherol) antioxidants were further determined in nematode infected plants following the addition of bacterial strains. The upregulation of photosynthetic activities were depicted by evaluating plant pigments and gas exchange attributes. An increase in the levels of phenolic compounds (total phenols, flavonoids, anthocyanins), osmoprotectants (total osmolytes, carbohydrates, reducing sugars, trehalose, proline, glycine betaine, free amino acids) and organic acids (fumaric, succinic, citric, malic acid) were reflected in infected plants, showing further enhancement after application of biocontrol agents. The study revealed the understanding of plant metabolism, along with the initiative to commercially exploit the biocontrol agents as an alternative to chemical nematicides in infected fields for sustainable agriculture.

## 1. Introduction

*Lycopersicon esculentum* Mill. is one of the most important crops of the world that is mostly cultivated by the farmers after potato due of its broader adaptability, high yield and its suitability for number of uses in fresh and processed food markets [1]. It is considered to be a high nutrient value crop due to its abundant health benefits, as it is comprised of higher vitamin C, flavonoids, carotenoids (β-carotenes), folate, water soluble antioxidants, and a range of phytochemicals that are effective against cancer and many other deadly diseases [2].

Due to sky-rocketed world population, it is very challenging to maintain the standard and productivity of food supplies for the entire population. It has been recently reported that, productivity of the tomato crop has dwindled worldwide, due to amyriad of diseases caused by pests and pathogens, specifically plant-parasitic nematodes (PPNs) [3]. The PPNs plummet nearly about 100 billion dollars of yearly agricultural losses, in spite of using all the commercially available methods for nematode management [4]. This has been mediated by fluctuation in the global temperatures caused by climatic factors, favouring PPNs population in soil either by affecting their life cycle or altering the host machinery and facilitating the infection process to take place [5].

The PPN, *Meloidogyne incognita* is a microscopic, soil inhabiting nematode that mainly feeds on living plant cells [6]. The J2 (second stage juvenile) is the infectious stage, that enters into the plant via roots and migrate across intercellular spaces towards vascular tissues [7]. After getting effectively established within the plant tissues, they migrate towards the nearby cells and initiate the formation of permanent feeding sites. The feeding cells or sites are composed of giant cells (multinucleate) that mature to form recognisable knots or galls onto root surfaces [8]. These are the regions within which PPNs proliferate and develop, thereby, launching an interactive association with the plants [7,9]. The PPN releases effector proteins from stylet to initiate the parasitic relationship with plants in for maintaining the feeding sites [10]. PPNs modifies the plant cell wall, host protein machinery and defense proteins, leading to protein denaturation and transcriptional changes in the different metabolic processes of plants [11]. The symptoms show the formation of root galls, hypertrophic or hyperplasic roots, disturbance in water and mineral transport, and inhibition in water-nutrient balance that leads to chlorosis and disrupted photosynthetic attributes of plants [12]. Moreover, they can also provoke morphological alterations of feeding cells, enlargement of nuclei, condensation of cytoplasmic bridges, disorientation of vacuoles and proliferation of other organelles such as plastids and mitochondria [13]. In addition, they enhance the susceptibility of infected plants towards other pathogens such as *Fusarium,* etc. [14].

At present, the management strategies employ the consumption of synthetically derived chemical nematicides to control the PPN proliferation in the fields or practising resistant plant varieties [15,16]. However, taking into consideration the harsh effects of nematicides on environment as well as flora and fauna, their use have been noticeably reduced [17]. Other strategies include the use of crop resistant varieties and crop rotation methods, which come with many limitations [3]. Therefore, the present scenario provides an impetus to look for an alternative and environmental friendly practice for controlling PPNs.

Plant growth promoting rhizobacteria (PGPR), a ubiquitous group of micro-organisms inhabiting rhizosphere have the potential to be greatly used as biocontrol agents [18,19]. They confer many vitalfunctions to the host plants such as releasing various phytohormones (Indole-3-acetic acid (IAA), ethylene, ABA, gibberellins, salicylic acid, cytokinins), enzymes (ACC (1-aminocyclopropane-1-carboxylate) -deaminase, chitinases, glucanases etc.), phosphate solubilization, siderophore production, nitrogen fixation and protection towards different pathogenic organisms including PPNs [20,21]. The PGPR mediated plant resistance towards PPNs is provided by production of various antagonistic compounds, lytic enzymes, toxins and antibiotics that inhibit the nematode proliferation or directly killing them [22]. Moreover, PGPR also colonises the plant roots in the rhizosphere, produces various metabolites and their by-products that either affect the motility of PPN, or their emergence and penetration within the plant roots [23]. In addition, they also protect plants through ISR (induced systemic resistance) and synthesising quorum sensing molecules (for e.g., acylhomoserine lactone) toregulate the activities of nematodes in the rhizosphere [24,25]. Several PGPR genera that have been used as biocontrol agents include *Azospirillum, Azotobacter, Pseudomonas, Gluconacetobacter, Azoarcus, Bradyrhizobium, Burkholderia, Bacillus, Alcaligenes, Paenibacillus, Serratia* etc. [26,27,28,29]. Furthermore, PGPR also comes up with the interior functions of the plants such as maintaining the ROS levels generated inside plant through effective scavenging mechanisms [16,30]. They also trigger the antioxidant levels in plants (enzymatic and non-enzymatic) during nematode infection, a prerequisite for triggering defense responses within them [31]. The PGPR have also been significantly known to modulate the levels of plant metabolites by up-regulating the enzymatic activities of secondary metabolites such as phenols, sugars, amino acids and organic acids [23,26]. In addition, PGPR were found to induce ISR in tomato plants infested with nematodes through modulating the biochemical profiles of plants [26]. The various biochemical analysis revealed that host plant develops resistance against PPN via activating or de-activating of certain enzymes involved in physiological and biochemical processes of plants [23].Especially, the strains from genera *Pseudomonas* and *Burkholderia* have been used as the biological control agents for PPNs in *L. esculentum,* as observed in our previous studies conducted on 10-days old seedlings [32]. It was reported that PGPR strains led to improved tomato resistance from PPN infection through activation of different antioxidant mechanisms with plants that directly scavenged the free radicals generated in response to infection [32].

The present research was carried out as extension to our previous studies to explore the role of *Pseudomonas aeruginosa* (MTCC7195; M1) and *Burkholderia gladioli* (MTCC10242; M2) on 45-days old nematode infested *L. esculentum* plants raised under field conditions. The morphological parameters, plant pigments, gas exchange parameters, oxidative damage, enzymatic and non-enzymatic antioxidants, phenolic compounds, osmoprotectants and organic acids were assessed in the present study.

## 2. Materials and Methods

### 2.1. Biocontrol Agents

Two plant growth promoting rhizobacterial strains, *Pseudomonas* aeruginosa; MTCC7195 (M1) and *Burkholderia gladioli*; MTCC10242 (M2) were used as biocontrol agents in the present study. Strainswere purchased from IMTECH (Mohali, Punjab, India) in the lyophilized form, sealed in the culture tubes. They were revived in laminar air flow under aseptic conditions using 50 mL Nutrient broth media and placed in incubator with temperature conditions of 28 °C for 24–48 h. Later, they were sub-cultured time to time for further use. After that, 1 mL of broth culture was taken and inoculated into autoclaved 50 mL nutrient broth media and placed in incubator at specific conditions for bacterial proliferation. This was further spun in the centrifuge at 8,000 rpm for 10–15 min at 4 °C, until the pellet was formed. The supernatant was removed and pellet was washed thoroughly using double distilled water. After washing the pellet, it was suspended in double distilled water and 10^9^ cells/mL population was maintained.

### 2.2. Root-knot Nematode (Meloidogyne incognita) Culture

The *M. incognita* culture was raised and maintained in green house by collection of samples from infected sites. *M. incognita* was recognized, screened and isolated from the sites to use it further for experimental purpose. They were identified by assessing perineal patterns of females that are formed by expansion or alteration of juvenile bodies, followed by retained tail tips, lateral patterns or presence of phasmids [33]. The roots were washed and cleaned properly (0.9% saline solution) to remove the soil particles. The egg masses were carefully taken off from infected roots and suspended into 45% lactic acid solution. They were allowed to hatch in distilled water into second stage juveniles which were further used to inoculate the tomato and brinjal seedlings raised for maintaining the pure cultures. Second stage juveniles obtained from cultures were then used for experimentation.

### 2.3. Raising of Plants

Authoritative seeds of *L. esculentum* Mill. (var. Pusa Ruby) were purchased from market, sterilized by 0.01% mercuric chloride and rinsed for 4, 5 times with double distilled water. Soil was prepared by mixing garden soil, sand and organic manure in 3:1:1 ratio followed by autoclaving. Pots were washed and cleaned thoroughly and filled with 300 g soil mixture to which the seeds were sown. After the appearance of true leaves, inoculation of *M. incognita* was given near roots (300 J2s). Later, the soil was given rhizobacterial treatment after the onset of germination and inoculation of *M. incognita.* The pots were marked and kept under field conditions and irrigated properly. The harvesting was done carefully after 45-days of inoculation/germination by washing the roots with distilled water to get rid of soil particles. The plants were stored to carry out morphological, physiological and biochemical investigations.

### 2.4. Growth Attributes

The growth attributes of 45-days old *L. esculentum* plants were measured through assessing root length and shoot length. Further, plant biomass was also determined in terms of fresh weight and dry weight of samples.

### 2.5. Photosynthetic Pigments

#### 2.5.1. Total Chlorophyll and Carotenoid Content

The estimation of total chlorophyll and carotenoid content was done by Arnon [34] and Maclachlan and Zalik [35] method. For this, 1 g sample was grounded in 80% acetone, followed by centrifugation in cooling centrifuge with 4 °C at 10,000 rpm for 15 min. After that, absorbance of supernatant was read at 645 and 663 nm respectively for total *chl* and 480 and 510 nm for carotenoid content.

#### 2.5.2. Total Xanthophyll Content

Estimation of total xanthophyll content was done by Lawrence [36] protocol. For this purpose, 50 mg plant sample completely dried and crushed was taken in the flask. The extractant (30 mL) was prepared by combining hexane (10 mL): absolute alcohol (6 mL): toluene (7 mL): acetone (7 mL) and transferred and meticulously mixed for 15 min. To this, 2 mL 40% Met. KOH was mixed and heated to 58 °C in water bath. Sample incubation was done under dark conditions for an hour after which 30 mL of hexane in addition to 10% Na_2_SO_4_ was combined in flask to make it to 100 mL volume. Again, incubations for an hour were done under dark conditions after vigorously mixing the contents. Later, the upper layer was removed to other flask and hexane was added to make final contents of 50 mL after which the optical density at 474 nm was read.

#### 2.5.3. Gaseous Exchange Parameters

The measurement of gas exchange attributes were done in 45-days old plants. Estimation of Net photosynthetic rate (Pn), Transpiration rate (E), Stomatal conductance (Gs) and intercellular CO_2_ rate (Ci) was done by Portable Photosynthesis Measuring System Unit (Li COR-6400, LiCOR Instruments, Lincoln, NE, USA) during sunny day. Maintenance of relative humidity (80–90%); temperature (25 °C); photon flux density (1000 µmol m^−2^ g^−1^) and CO_2_ rate (400 µmol mol^−1^) were the essential conditions for the standard working of the instrument.

### 2.6. Oxidative Burst

#### 2.6.1. Superoxide Anion Content

The measurement of superoxide anion content was done after following Wu et al. [37] method. Crushing of 1 g plant material in 2% polyvinylpyrrolidone (PVP) and 0.5% Triton X-100 containing, 4 mL 50 mM phosphate buffer (pH-7.8) was done. Then, they were spun at 10,000 rpm for 15 min. After removing the 0.5 mL supernatant in separate tube, 0.5 mL of phosphate buffer and 0.1 mL hydroxylamine hydrochloride was mixed. Incubations at 25 °C for 30 min were done. To this, 1 mL each of 1-naphthylamine and 3-aminobenzenesulphonic acid was supplemented and placed for 15 min at 26 °C. Optical density at 530 nm was further read.

#### 2.6.2. Hydrogen Peroxide Content

The estimation of hydrogen peroxide content by Velikova et al. [38] proposed method was done. Homogenisation of 100 mg of plant tissue in freshly prepared 0.1% Trichloroacetic acid (TCA) was done in cold motor pestle. They were then subjected to centrifuge at temperature of 4 °C, at 10,000 rpm for 20–25 min. The supernatant was removed in the separate tube and mixed with 0.5 mL of potassium phosphate buffer and 0.5 mL KI. Optical density at 390 nm was then recorded.

#### 2.6.3. Malondialdehyde Content

Estimation of malondialdehyde (MDA) accumulation was conducted by Heath and Packer [39] method. To start with the measurement, 1g of fresh plant material was macerated using freshly prepared 0.1% 5 mL TCA. Samples were centrifuged at 8000 rpm at 4 °C for 15 min. 1 mL supernatant was removed in a separate tube to which 4 mL of 0.5% thio-butyric acid (TBA) (made with 20% TCA) was combined. The incubations for 30–35 min at 90 °C were given by placing the tubes in water bath. After cooling the tubes, the optical density at 532 nm was read.

### 2.7. Antioxidative Enzymes

Specific activities of various antioxidative enzymes were evaluated by crushing 1 g plant tissue in 4 mL 100 mM potassium phosphate buffer (pH7.0). Preparation of potassium phosphate buffer was done by combining four different ingredients i.e., 1 mM EDTA, 5% polyvinyl polypyrrolidone, 2 mM phenylmethylsulfonyl fluoride and 0.5% triton-X-100. The crushed material was allowed for centrifugation for 20 min at 10,000 rpm.

#### 2.7.1. Superoxide dismutase Activity (EC.1.15.1.1)

Specific activity of superoxide dismutase (SOD) was calculated by using standard protocol [40]. The ingredients of the reaction were 50 mM Na_2_CO_3_ buffer, 1 mM hydroxylamine hydrochloride (pH6), 0.03% Triton-X 100, 24 µM NBT, 0.1 Ethylenediaminetetraacetic acid (EDTA) and plant extract. The rise in absorbance at 540 nm was further noted.

#### 2.7.2. Guaiacol peroxidase Activity (EC. 1.11.1.7)

Specific activity of guaiacol peroxidase (POD) was estimated by Pütter [41] method. The ingredients of the reaction were potassium phosphate buffer (20 mM with pH7.0), freshly prepared guaiacol solution (10 mM), 124 mM H_2_O_2_ and plant extract. The increase in absorbance was recorded at 436 nm.

#### 2.7.3. Catalase Activity (EC.1.11.1.6)

Specific activity of catalase (CAT) was examined according to Aebi [42] procedure. The ingredients of the reaction were potassium phosphate buffer (50 mM with pH7.0), H_2_O_2_ (20 mM) and plant extract. H_2_O_2_ dismutation declined optical density that was read at 240 nm.

#### 2.7.4. Glutathione Peroxidase Activity (EC. 1.11.1.9)

Specific activity of glutathione peroxidase (GPOX) was measured by procedure given by Flohé and Günzler [43].The components of reaction were phosphate buffer (50 mM; pH 7.0), Glutathione (0.15 mM), EDTA (0.5 mM), hydrogen peroxide(0.15 mM), NaN_3_ (0.15 mM), 2.5 units/mL glutathione reductase (GR), NADPH (0.15 mM) and plant extract. Absorbance at 340 nm was read.

#### 2.7.5. Ascorbate Peroxidase Activity (EC.1.11.1.11)

Specific activity of ascorbate peroxidase (APOX) was calculated by process suggested by Nakano and Asada [44]. The ingredients of reaction were ascorbate (0.5 mM), potassium phosphate buffer (50 mM) with pH 7.0, hydrogen peroxide (2 mM), and plant extract. Optical density at 290 nm was read.

#### 2.7.6. Dehydroascorbate Reductase Activity (EC. 1.8.5.1)

Specific activity of dehydroascorbate reductase (DHAR) was evaluated by procedure propounded by Dalton et al. [45]. The reaction contained following ingredients; 50 mM potassium phosphate buffer with pH7, dehydroascorbate (0.25 mM), glutathione reduced (2.5 mM) and plant extract. Optical density at 265 nm was then read.

#### 2.7.7. Glutathione-*S*-Transferase Activity (EC.2.5.1.18)

Specific activity of glutathione-*S*-transferase (GST) was quantified by method offered by Habig and Jakoby [46]. Reaction was initiated by combining 20 mM potassium phosphate buffer with pH7.5, glutathione reduced (20 mM), cDNB (15 mM) and plant extract. Optical density at 340 nm was noted.

#### 2.7.8. Glutathione Reductase Activity (EC.1.6.4.2)

Specific activity of GR was estimated by process presented by Carlberg and Mannervik [47]. The ingredients of reaction were 20 mM potassium phosphate buffer with pH7, 1 mM GSSG, 1 mM EDTA, 0.2 mM NADPH and plant extract. After combining all, absorbance at 340 nm was taken.

#### 2.7.9. Polyphenol oxidase Activity (EC. 1.14.18.1)

Specific activity of polyphenol oxidase (PPO) enzyme was measured by Kumar and Khan [48] method. The reaction ingredients were 50 mM potassium phosphate buffer with pH6, 80 mM catechol and enzyme extract. They were combined and sited for 5–10 min, after which H_2_SO_4_ (3.5 N) was added. Absorbance at 495 nm was then recorded.

### 2.8. Non-Enzymatic Antioxidants

To estimate the levels of non-enzymatic antioxidants, fresh plant tissue was grounded in 50 mM of 4 mL Tris Buffer with pH10 using cold, sterile and autoclaved motor pestle. After that, centrifugation in cold centrifuge (4 °C) at 10,000 rpm was done for 15–20 min. Collection of supernatant in other tubes was done to quantify antioxidants (glutathione, ascorbic acid and tocopherol).

#### 2.8.1. Glutathione Content

Estimation of glutathione content was done through standard protocol offered by Noctor and Foyer [49]. For this, combination of following contents, l00 µL supernatant, 50 µL DTNB, 4 mL absolute alcohol and l000 µL Tris buffer were taken in a tube and placed under room temperature for 10 min. The tubes were vortexed for 10 min until a yellow coloured product is formed. Optical density at 412 nm was read.

#### 2.8.2. Ascorbic Acid Content

Estimation of ascorbic acid was conducted through procedure opted by Roe and Kuether [50]. For this, combination of 1 mL supernatant, 1 mL freshly prepared 50% TCA and 0.5 mL DPNH was done. The tubes were shaken vigorously after which they were incubated at room temperature for 4 h. The osazones crystals produced were dissolved in cold sulphuric acid. Incubation in ice for 20 min was given and absorbance at 540 nm was read.

#### 2.8.3. Tocopherol Content

Estimation of tocopherol content was done by Martinek [51] established protocol. For this, 500 µL homogenate was combined to 500 µL distilled water, 500 µL absolute ethanol prepared by adding 0.12% FeCl_3_6H_2_O. The tubes were shaken vigorously until the protein crystals were developed. Crystals were dissolved in 500 µL xylene and vortexed for 2 min. The layer formed on upper phase was combined to TPTZ in separate tube and optical density at 600 nm was read.

### 2.9. Phenolic Compounds

#### 2.9.1. Total Phenols

Estimation of total phenol content was conducted through method examined by Singleton and Rossi [52]. For this, 500 mg oven dried sample was grounded in 60% ethanol, after which it was warmed at 65 °C using water bath for 20 min. Samples were filtered by Whatmann (GE Healthcare Life Sciences, Maidstone, UK) filter paper till the residue was left behind. 100 mL of total sample volume was maintained by 60% ethanol. There was 2 mL of this sample mixed in another tube containing 8 mL FC reagent and 8 mL sodium carbonate. Incubation for 4 h was given followed by measuring optical density at 765 nm. (Standard curve prepared by gallic acid).

#### 2.9.2. Total Flavonoids

Estimation of total flavonoids was conducted by standard procedure given by [53]. For this, 100 mg of dried sample was macerated in 4 mL absolute alcohol. The preparation of extractant was done by combining 3 mL each of 5% NaNO_2_, 5% AlCl_3_ and distilled water. 1 mL of above prepared extractant was then combined to 2 mL sodium hydroxide and 2.5 mL of distilled water in sample tube. Incubation was done for 10 min followed by measuring optical density at 510 nm (Standard used: Rutin).

#### 2.9.3. Total Anthocyanins

Estimation of total anthocyanins was done through procedure followed by Mancinelli [54]. For this, 1000 mg of plant material was crushed in reagent prepared by combining absolute methanol, distilled water and hydrochloric acid in the ratio of 78:20:2. The sample was incubated under cold conditions (4 °C) for 12 h and spun at 14,000 rpm for 20 min. Absorbance at 530 and 657 nm was read to calculate the anthocyanin content.

### 2.10. Osmoprotectants

#### 2.10.1. Total Osmolytes

Estimation of total osmolytes was done with Vapour Pressure Osmometer (Vapro 5600, ELITechGroup, Paris, France). The fresh plant samples were stored in liquid nitrogen and thawed for sap extraction using syringe. The sap was collected for further analysis. Only 10 μL of sap was used for analysis and loaded directly on filter paper discs kept upon the instrument. Calibration of instrument was done using Sodium chloride standard kits with 1000, 290 and 100 mOsm osmolarities that come along with the instrument. Values displayed on the instrument were noted after maintaining room temperature conditions of 22, 23 °C.

#### 2.10.2. Total Carbohydrates

Total carbohydrates were calculated through the Hedge et al. [55] procedure. For this, 100 mg sample was warmed in 4 mL of 2.6 N HCl for 5 h. Incubations at room temperature were given followed by the addition of sodium carbonate to offset the reaction. A total volume of 30 mL was maintained by addition of 25 mL distilled water. There was 4 mL of anthrone reagent taken in the separate tube to which 1 mL of above sample was added and subjected to boil for 15 min. Samples were cooled and optical density at 630 nm was read (d-glucose: standard).

#### 2.10.3. Total Reducing Sugars

The analysis of total reducing sugars was done by method provided by Miller [56]. For this, 100 mg dried and finely powdered sample was crushed in 80% absolute alcohol. Preparation of DNSA was done through combination of 0.2 g phenol crystals and 0.05 g Na_2_SO_3_ under cold conditions. In total, 3 mL of this freshly prepared reagent was added to 1 mL of above supernatant and 40% KNaC_4_H_4_O_6_·4H_2_O. Incubation for 2 min was given at room temperature to record the optical density at 510 nm.

#### 2.10.4. Trehalose Content

Estimation of trehalose content was done through Trevelyan and Harrison [57] assay. The analysis was begun by crushing 500 mg oven dried sample in 80% ethanol and further centrifuged under cold conditions of 4 °C at 10,000 rpm for 20 min. The components of reaction were combined by adding 100 μL of above extractant, 4 mL anthrone reagent and 3 mL freshly prepared TCA. Optical density at 620 nm was read after the formation of deep yellow coloured product (d-glucose: standard).

#### 2.10.5. Glycine Betaine Content

The measurement of glycine betaine (GB) content was predicted by Grieve and Grattan [58] protocol. To begin with the analysis, 500 mg dried sample was crushed in 0.05% toluene and 4 mL double distilled water which was then incubated for 24 h. Samples were filtered through filters with 0.2 µm pore size. The reaction components were mixed by combining 500 µL supernatant, 1000 µL 3 N HCl and 100 µL potassium iodide. Incubations of samples were done in ice for 4 h. To the reaction tubes, 3 mL chilled water and 8 mL 1,2-dichloroethane was mixed. The tubes were vortexed till the formation of two different layers. The above layer was thrown, and bottom pink layer was kept for analysis. Optical density at 365 nm was then read (Standard: betaine hydrochloride).

#### 2.10.6. Proline Content

Estimation of proline content was done through Bates et al. [59] analysis method. For this, 500 mg sample was crushed in 10 mL 3% sulphosalicylic acid. Centrifugation of sample tubes at 10,000 rpm for 15–20 min was done. Sample tubes containing 2 mL supernatant was separated and combined to 3 mL ninhydrin reagent and 3 mL glacial acetic acid. The tubes were heated to 100 °C and placed in ice after 5 min to counterbalance the reaction. Later, 5 mL toluene was further mixed to it and tubes were vortexed for 3 min. The red toluene layer was formed of which the optical density at 520 nm was read (Standard: l-proline).

#### 2.10.7. Free Amino Acid Content

Estimation of free amino acid content was done via Lee and Takahashi [60] method. To begin with, 100 mg dried sample was crushed using 4 mL absolute ethanol and warmed at 50 °C for 10 min using water bath. The reaction tubes were vortexed for 5 min. Later, freshly formed ninhydrin reagent was combined with 200 µL sample and heated for 5 min. Optical density at 570 nm was taken after the formation of blue colour.

### 2.11. Organic Acid Profiling

Organic acid profiling was done through following Chen et al. [61] method with slight modifications. To begin with the analysis, 50 mg dried crushed sample was taken to which 500 µL absolute methanol and 500 µL 2.5 N HCl was pooled. Sample tubes were then placed in shaker for 5 h, and centrifuged under cold conditions (4 °C) at 10,000 rpm for 10 min. The residue was left behind and above liquid was transferred into another tube to which 0.3 mL methanol and 0.1 mL 50% conc. sulphuric acid was combined, vortexed and incubated for 10 h at 65 °C in water bath. Sample tubes were cooled at 28 °C and 800 µL chloroform and 400 µL double distilled water mixed into it. Vortexing was done till the formation of two different layers. The basal layer was collected for organic acid analysis by GC-MS. The system (Shimadzu GC-MS-Q2010Plus, Japan) required 2 µL sample for analysis. Standard conditions of the instrument were: (i) for GC: helium carrier gas, column with flow rate of 1.7 mL/min, analytical column with length of 30 m, ID-0.025 mm and DB, 5 ms, temperature of 125 °C and 25 °C/min; (ii) for MS: ion source with temp 200 °C and detection mode is relative. The organic acid quantification was carried out by comparing mass spectra or peaks with National Institute of Standard and Technology and Wiley 7 Library.

### 2.12. Statistical Analysis

The morphological, physiological and biochemical data was statistically tested through the self-built program in Microsoft Excel (Microsoft Office Excel 2007, Albuquerque, New Mexico, USA). Results in the form of mean ± standard deviation (SD) (level of significance checked at *p* ≤ 0.05 and 0.01) were presented. Null hypothesis (H_0_) was verified by two-way ANOVA (analysis of variance). Data was also confirmed with Tukey’s multiple comparison test and HSD (honestly significant difference).

## 3. Results

### 3.1. Effect of Biocontrol Agents on Growth Parameters of PPN Infected L. esculentum Plants

The ramifications of microbial strains (*P. aeruginosa* (M1) and *B. gladioli* (M2) on morphological parameters of 45-days old *L. esculentum* plants raised under PPN infestation was investigated in terms of root length, shoot length, biomass (fresh weight, dry weight) and number of galls. The results indicated a remarkable reduction in the root length and shoot length of plants by 43.2 and 21.5% relative to control plants. The decline in the plant biomass of infected plants (fresh weight (35.1%) and dry weight (47.6%)) was also reported. However, after the inoculation of *P. aeruginosa* (M1) the enhancement in the root length, shoot length, fresh weight and dry weight was noted by 73.3, 54.3, 33.2, and 105.4%, respectively. The supplementation of *B. gladioli* (M2) in infected plants also maximised the root length (51.6%), shoot length (69.7%), fresh weight (49.6%) and dry weight (110.5%) when compared to PPN experimental plants. Furthermore, the number of galls were also measured in PPN inoculated plants. The gall numbers (27) were counted in plants inhabited with nematodes, and their significant reduction was noted in the plants supplemented with rhizobacterial strains. Reduction of 28.06% was observed after *P. aeruginosa* (M1) addition while reduction to 19.5% was seen after *B. gladioli* (B2) treatment in contrast to experimental controls (Table 1).

### 3.2. Effect of Biocontrol Agents on Photosynthetic Pigments of PPNPPN Infected L. esculentumPlants

**Figure 1:**
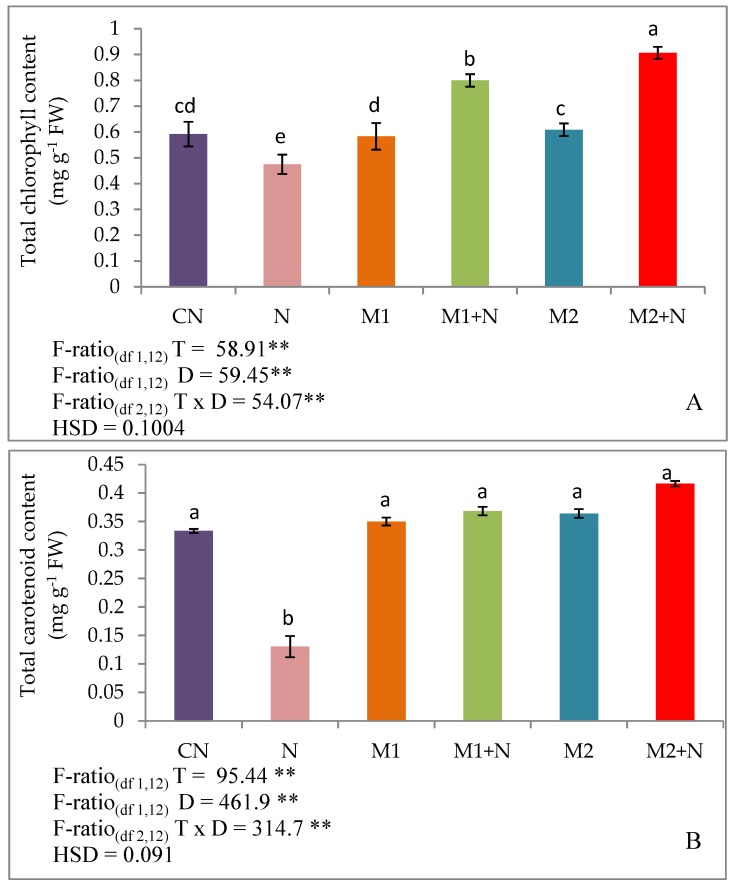

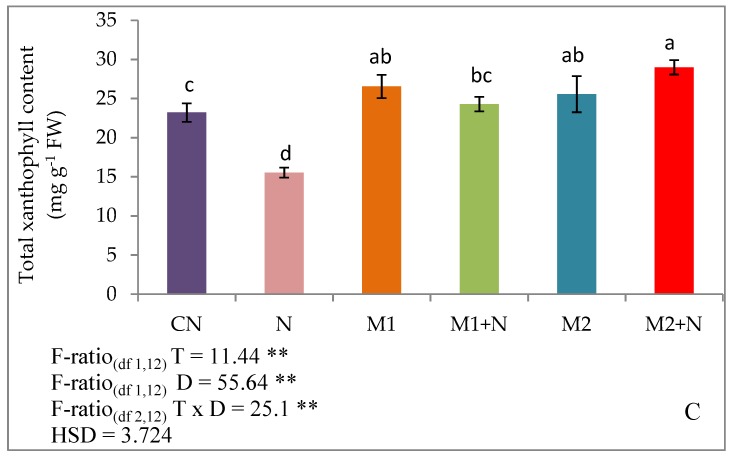
Effect of M1 (10^9^ cells/mL) and M2 (10^9^ cells/mL) on photosynthetic pigments (**A**) Total Chlorophyll content (**B**) Total Carotenoid content (**C**) Total Xanthophyll content in 45-days old *L. esculentum* under PPN infection. Data is presented as means of three replicates ± SD and honestly significant difference (HSD) values. F ratio values, * indicates significance at *p* ≤ 0.05 and ** indicates significance at *p* ≤ 0.01). Different letters on the table indicate that mean values of treatments are significantly different at *p* < 0.5 according to Tukey’s multiple comparison test.

The plant pigments were determined in terms of total chlorophyll, carotenoid and xanthophyll. It was evaluated that level of total chlorophyll was reduced to 19.7% in plants raised with nematode infestation as compared to experimental controls. But, supplementation of *P. aeruginosa* (M1) to nematode infected plants, led to uplifted levels of total chlorophyll from 19.7 to 68.5% respectively. Also, the inoculation of *B. gladioli* (M2) in infected plants stimulated the total chlorophyll content in plants by 91.1% respectively. The levels of carotenoids estimated, showed lowered levels by 60.9% in plants infected with nematodes relative to controls. After the application of *P. aeruginosa* (M1), a sharp enhancement in the level of carotenoids by 183% was noticed in nematode infested plants and by 220% after the inoculation of *B. gladioli* (M2). In addition, the levels of xanthophyll were also minimised by 33% in plants raised under nematode treated soils in contrast to control. A remarkable stimulation in xanthophyll content was measured by 56.4 and 86.7%, after the inoculation of *P. aeruginosa* (M1) and *B. gladioli* (M2) respectively, in nematode infested plants when compared with experimental controls (Figure 1).

### 3.3. Effect of Biocontrol Agents on Gas Exchange Parameters of PPN Infected L. esculentum Plants

The results depicted an alteration in the gas exchange parameters of plants raised under the influence of nematode infection and bacterial strains. The results displayed a noticeable reduction in net photosynthetic rate, stomatal conductance, intercellular CO_2_ and transpiration rate by 33.9, 31.6, 11.3 and 40.3% respectively, in 45-days old PPN infected plants. Supplementation of *P. aeruginosa* (M1) in infected plants led to an upregulation in all these gas exchange parameters. The inclination in the net photosynthetic rate, stomatal conductance, intercellular CO_2_ and transpiration rate was revealed by 32.7, 80.2, 8.64 and 30% respectively, after inoculations in the infected plants. The inoculation with *B. gladioli* (M2) also enhanced the activities of net photosynthetic rate, stomatal conductance, intercellular CO_2_ and transpiration rate by 87, 88.5, 24.4 and 46% respectively, in infected plants in comparison to experimental controls (Table 2).

### 3.4. Effect of Biocontrol Agents on Oxidative Damage in PPN Infected L. esculentum Plants

**Figure 2:**
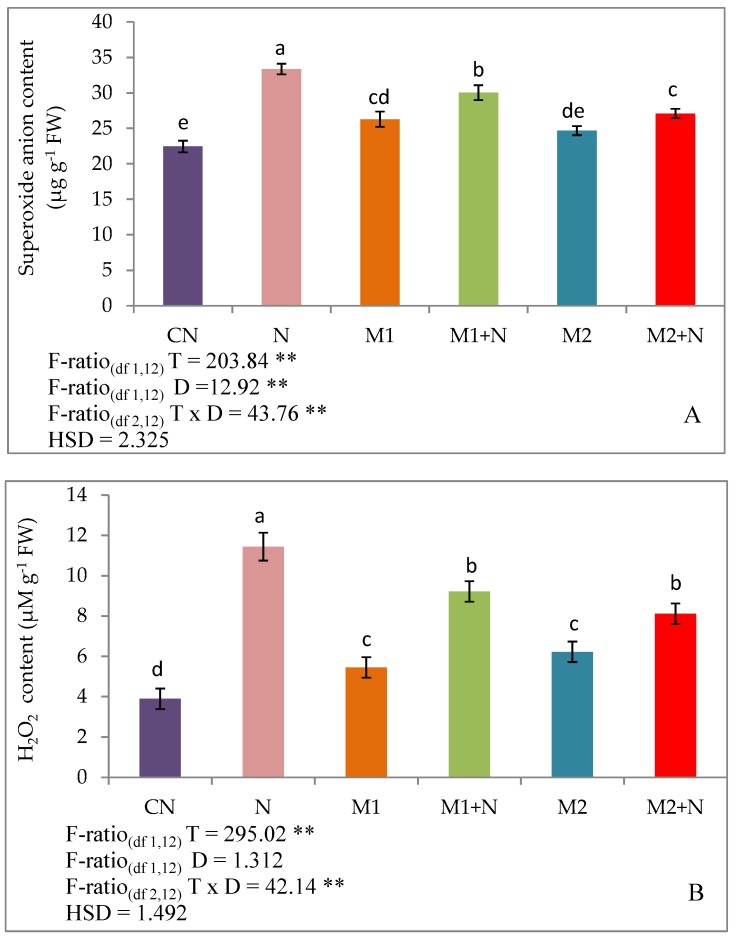

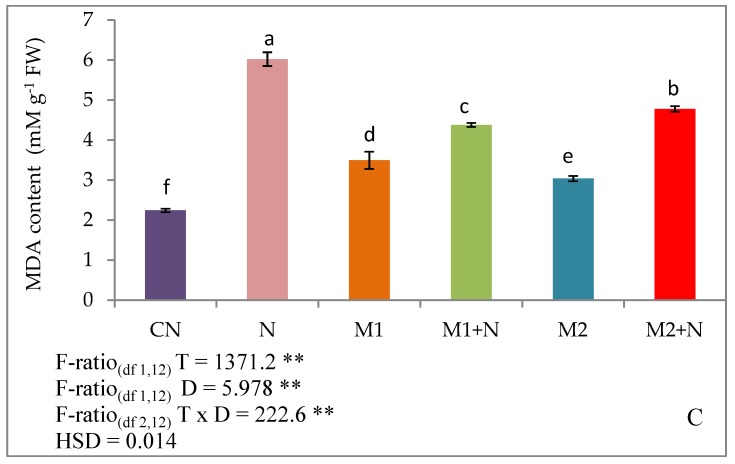
Effect of M1 (10 ^9^ cells/ ml) and M2 (10 ^9^ cells/ ml) on: (**A**) Superoxide Anion content, (**B**) Hydrogen peroxide content and (**C**) malondialdehyde (MDA) content in 45-days old *L. esculentum* plants under PPN infection. Data is presented as means of three replicates ± SD and HSD values. F ratio values, * indicates significance at *p* ≤ 0.05 and ** indicates significance at *p* ≤ 0.01). Different letters on the graphs indicate that mean values of treatments are significantly different at *p*< 0.5 according to Tukey’s multiple comparison test.

The oxidative damage was assessed by estimating the levels of oxidative stress markers namely, superoxide anion, H_2_O_2_ and MDA. It was revealed in the present investigation that PPN infestation in 45-days old plants inclined the levels of superoxide anion (48.7%), H_2_O_2_ (195.1%) and MDA content (168.7%) relative to infected control plants. The inoculation of *P. aeruginosa* (M1) waned off the superoxide anion levels by 9.98%, H_2_O_2_ levels by 19.4% and MDA levels by 27.3% in nematode infected *L. esculentum* plants. However, the amendment of PPN infected plants with *B. gladioli* (M2) also declined the superoxide anion, H_2_O_2_ and MDA levels by 18.7, 29.1 and 20.6%, respectively (Figure 2).

### 3.5. Effect of Biocontrol Agents on Antioxidative Enzymes Activities of PPN Infected L. esculentum Plants

The activities of different antioxidative enzymes; SOD, POD, CAT, GR, GST, APOX, GPOX, DHAR and PPO were measured in 45-days old nematode infected *L. esculentum* plants. The results showed an elevation in the activities of SOD (34.6%), POD (100.5%), CAT (31.1%), APOX (13.6%), GPOX (42.7%), DHAR (9.98%), GST (26.3%), GR (68%) and PPO (19.2%) in PPN infected plants as compared to untreated plants. The amendment of PPN infected plants with *P. aeruginosa*(M1) further upregulated the activities of SOD, POD, CAT and APOX by 17.3, 30.1, 10.82 and 15.48% respectively. Also, the activities of GPOX, GST, DHAR, GR and PPO were also further stimulated in infected plants on treatment with *P. aeruginosa*(M1) by 6.98, 38.5, 16.07, 1.74 and 64.4% respectively. Moreover, the augmentation of *B. gladioli* (M2) also triggered the SOD, POD, CAT and APOX by 9.4, 12.8, 7.83, 18.1% respectively in plants raised under PPN infection. Along with this, GST, GPOX, GR, DHAR and PPO were also recorded to be elevated by 61.8, 19.16, 7.29, 21.76 and 46.02% respectively in plants in the presence of *B. gladioli* (Table 3).

### 3.6. Effect of Biocontrol Agents on Non-Enzymatic Antioxidants of PPNInfected L. esculentum Plants

**Figure 3:**
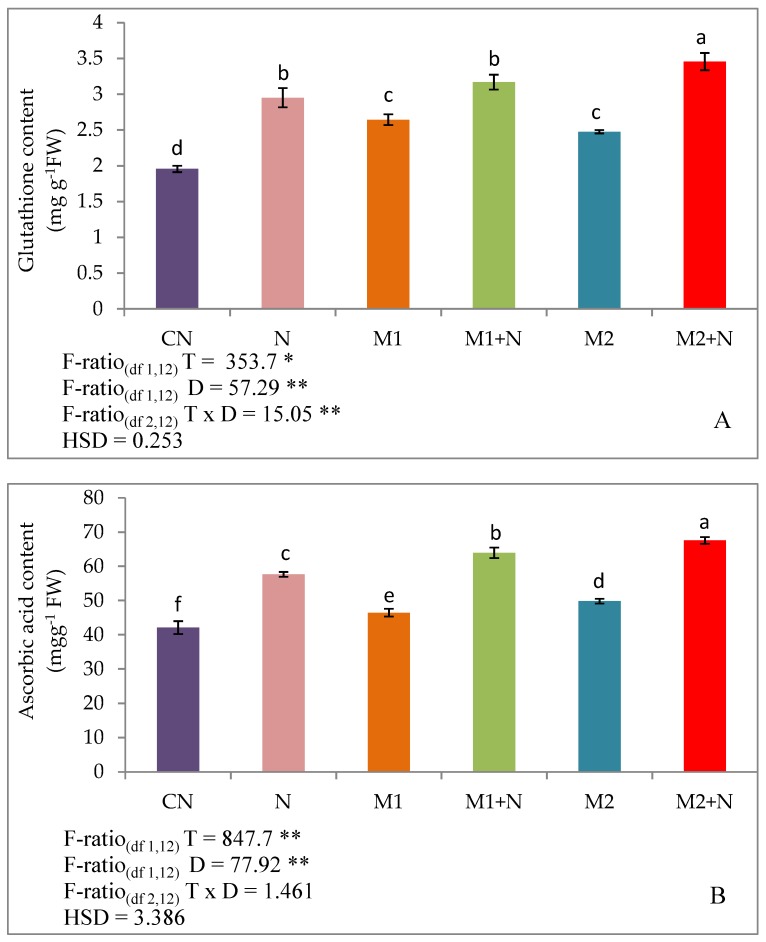

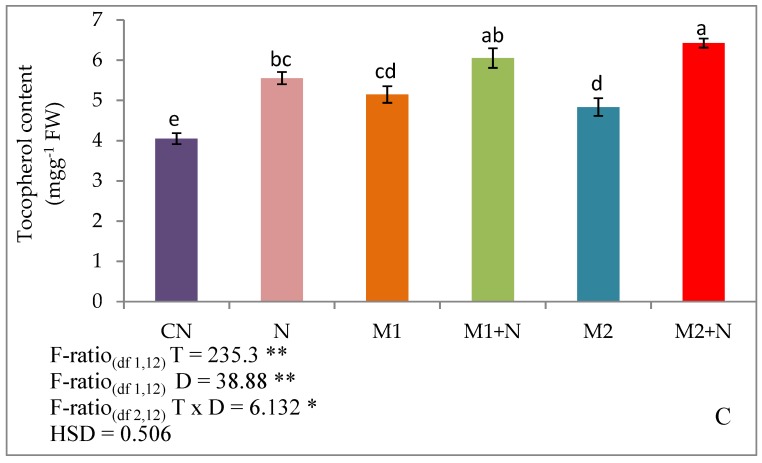
Effect of M1 (10^9^ cells/ ml) and M2 (10^9^ cells/ ml) on: (**A**) Glutathione content, (**B**) Ascorbic acid content and (**C**) Tocopherol content in 45-days old *L. esculentum* plants under PPN infection. Data is presented as means of three replicates ± SD and HSD values. F ratio values, * indicates significance at *p* ≤ 0.05 and ** indicates significance at *p* ≤ 0.01). Different letters on the graphs indicate that mean values of treatments are significantly different at *p*< 0.5 according to Tukey’s multiple comparison test.

The results pertaining to non-enzymatic antioxidants, glutathione, ascorbic acid, and tocopherol were found to be modulated in plants subjected to nematode infection and microbial treatment. A remarkable stimulation in the non-enzymatic antioxidants was estimated in the nematode infected plants. The glutathione content was raised by 51.2% in nematode infested plants relative to experimental controls. This was further accelerated after the addition of *P. aeruginosa* (M1) in infected plants and by 7.11 and 16.9% after the treatment of *B. gladioli* (M2) respectively. A similar observation was recorded for ascorbic acid levels where an escalation in the ascorbic acid levels was noted by 36.9% in infested plants while that of controls. This was further augmented by *P. aeruginosa*(M1) and *B. gladioli* (M2) independently, after which an enhancement in the ascorbic acid levels by 10.9% and 17.2% were reported. Another antioxidant, tocopherol was also estimated and found to be stimulated by 37.3% in PPN infested plants. Moreover, an inoculation of *P. aeruginosa*(M1) raised the tocopherol content in PPN affected plants by 9 and 15.67% by amendment with *B. gladioli* (M2) respectively (Figure 3).

### 3.7. Phenolic Compounds

#### Effect of Biocontrol Agents on Total Phenols, Flavonoids and Anthocyanins in PPN Infected *L. esculentum* Plants

**Figure 4:**
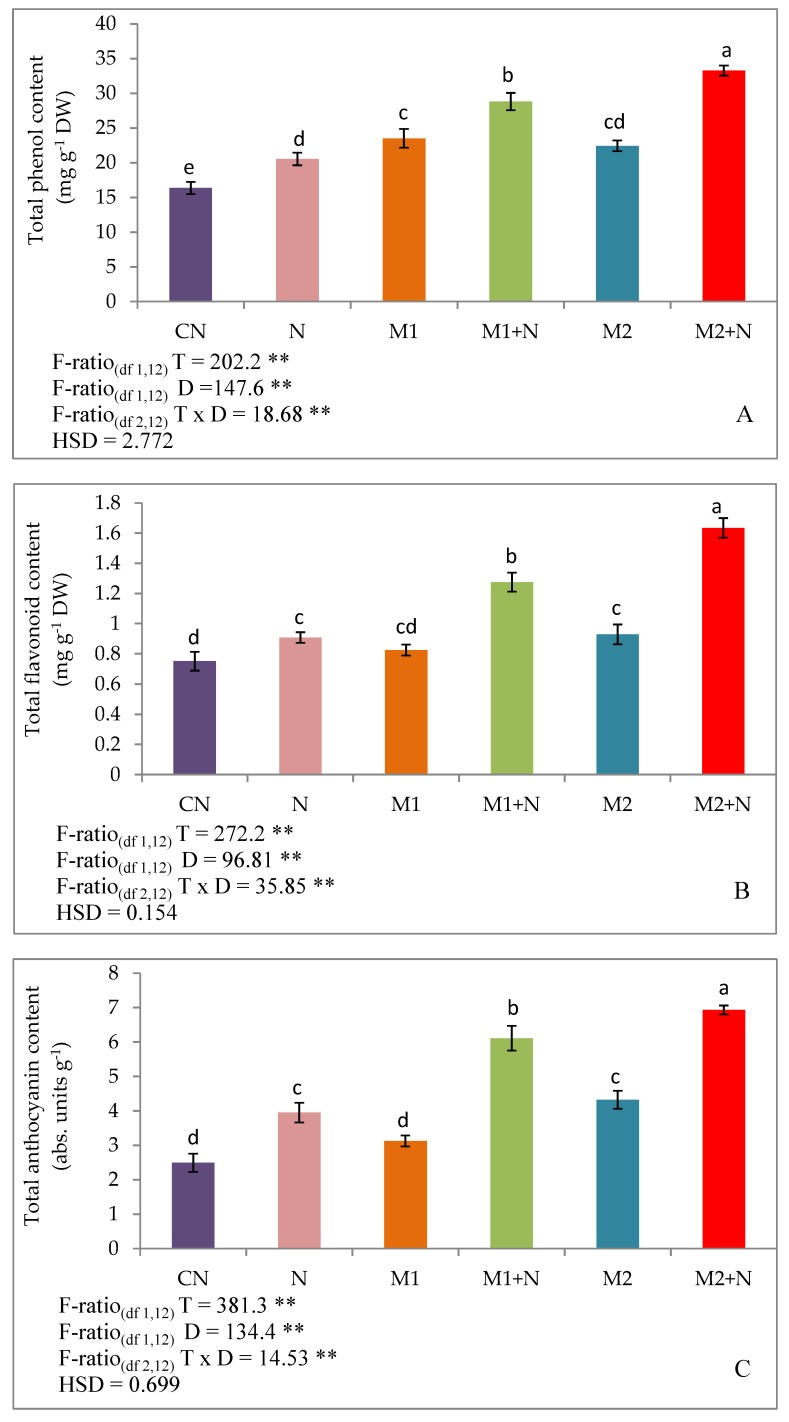
Effect of M1 (10^9^ cells/mL) and M2 (10^9^ cells/mL) on Phenolic compounds (**A**) Total Phenols (**B**) Total Flavonoids (**C**) Total Anthocyanins in 45-days old *L. esculentum* under PPN infection. Data is presented as means of three replicates ± SD and HSD values. F ratio values, * indicates significance at *p* ≤ 0.05 and ** indicates significance at *p* ≤ 0.01). Different letters on the table indicate that mean values of treatments are significantly different at *p*< 0.5 according to Tukey’s multiple comparison test.

The present study depicted that measurement of total phenols, flavonoid and anthocyanin levels were enhanced in the plants stressed with nematodes. The upliftment in the levels of total phenols occurred by 25.56%, flavonoids by 20.93% and anthocyanins by 58.46%, respectively. However, the further enhancement in the levels of these phenolic compounds were observed after the treatment of *P. aeruginosa* (M1) by 40.37% (total phenols), 40.46% (flavonoids) and 54.67% (anthocyanins) in infected plants. The inoculation of infected plants with *B. gladioli* (M2) also maximised the levels of total phenols, flavonoids and anthocyanins by 62.1, 14 and 75.51% in contrast to experimental controls respectively (Figure 4).

### 3.8. Osmoprotectants

#### 3.8.1. Effect of Biocontrol Agents on Total Osmolytes, Carbohydrates, Reducing Sugars in PPN Infected *L. esculentum* Plants

**Figure 5:**
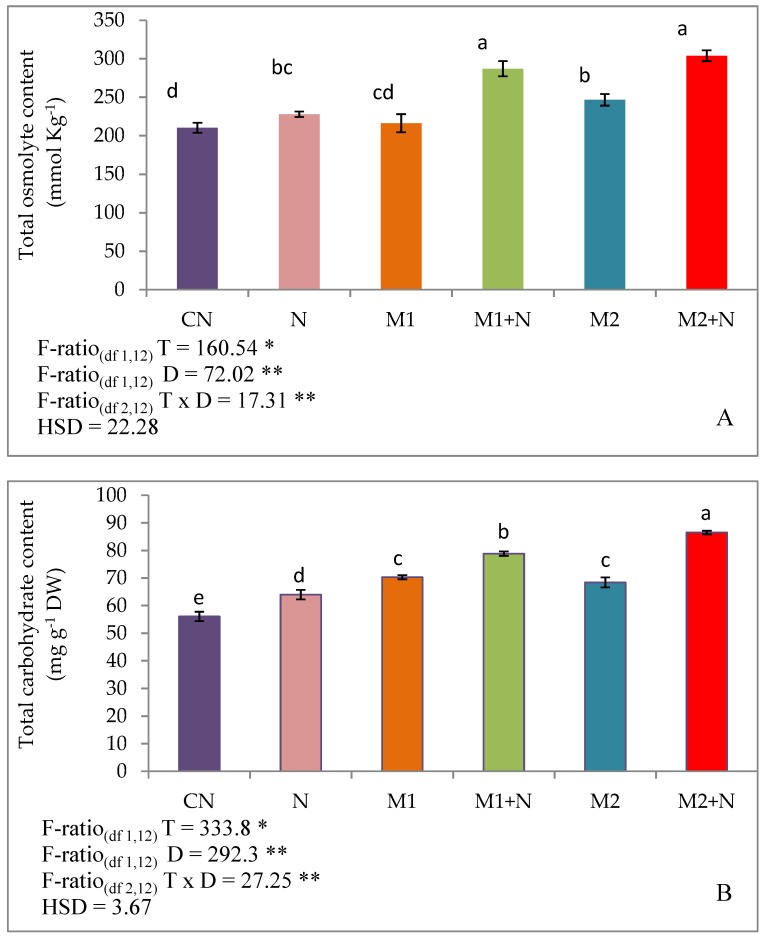

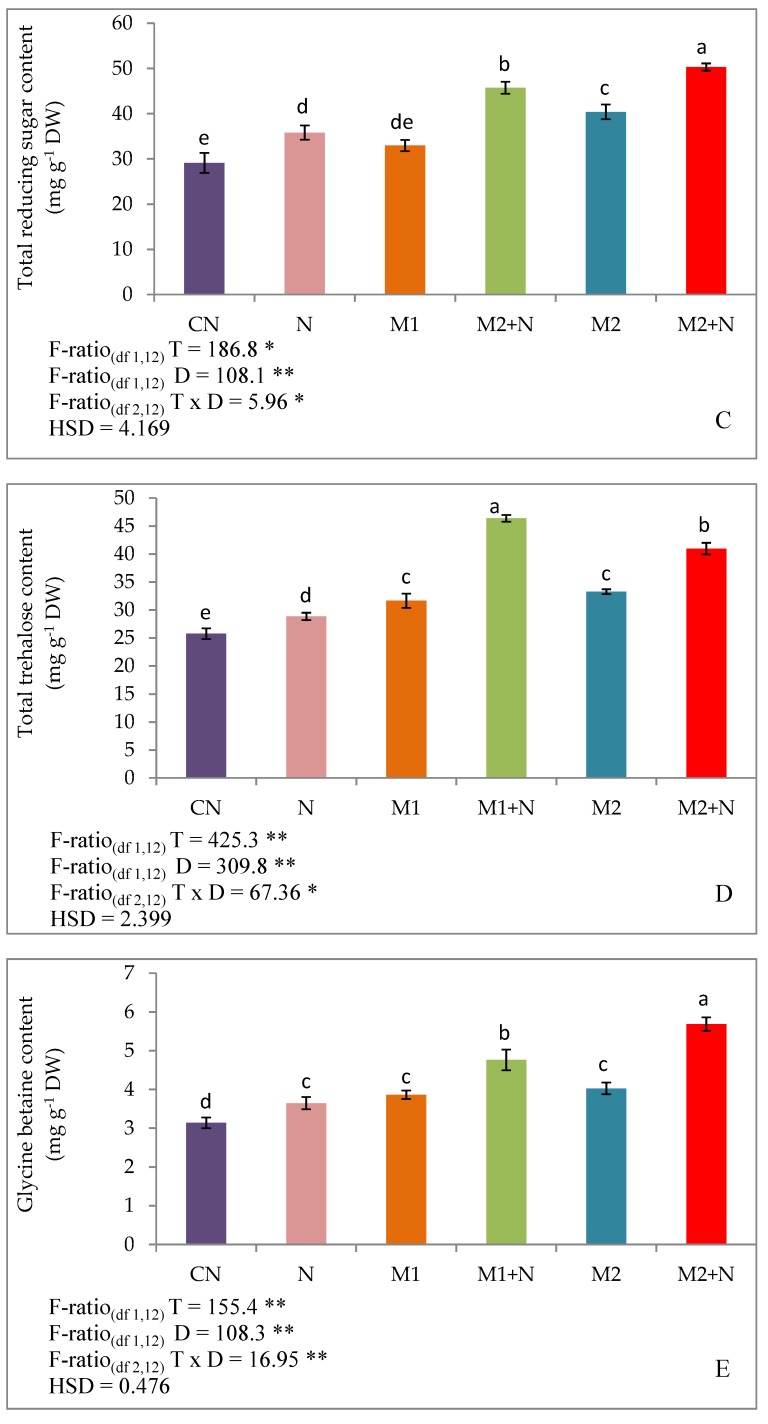

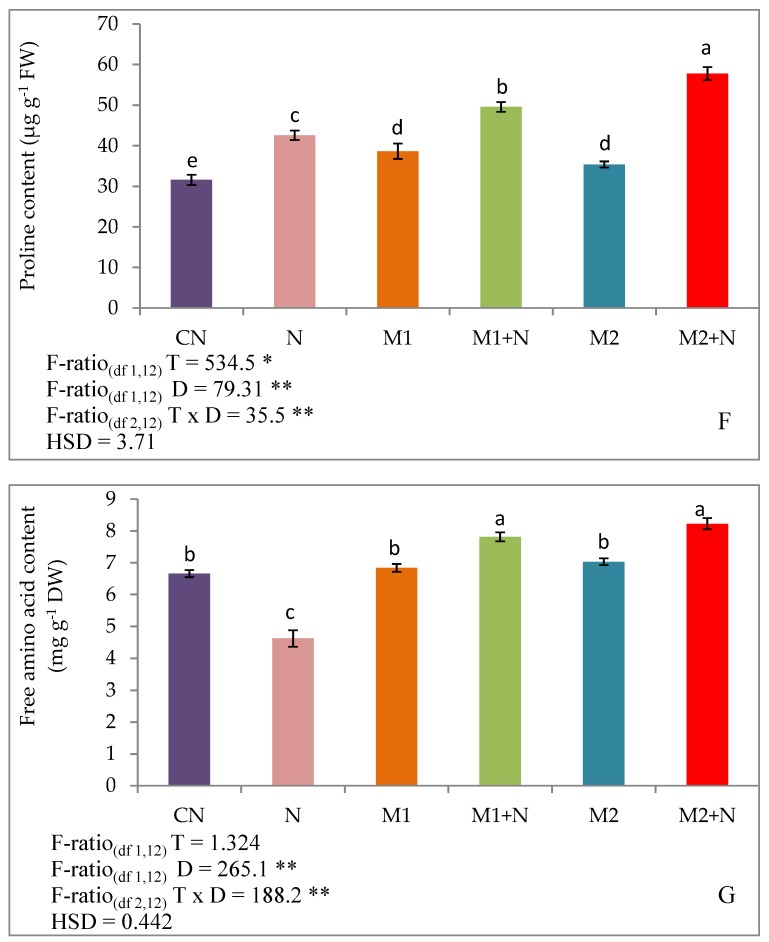
Effect of M1 (10^9^ cells/mL) and M2 (10^9^ cells/mL) on different Osmoprotectants (**A**) Total osmolytes (**B**) Total carbohydrates, (**C**) Total reducing sugars (**D**) Trehalose content (**E**) Glycine betaine content (**F**) Proline content (**G**) Free amino acid content in 45-days old *L. esculentum* under PPN infection. Data is presented as means of three replicates ± SD and HSD values. F ratio values, * indicates significance at *p* ≤ 0.05 and ** indicates significance at *p* ≤ 0.01). Different letters on the table indicate that mean values of treatments are significantly different at *p*< 0.5 according to Tukey’s multiple comparison test.

The levels of total osmolytes, carbohydrates and reducing sugars were uplifted by 8.38, 14.1 and 23.09% respectively, in PPN stressed plants in comparison to experimental control plants. Increase in the total osmolytes was recorded by 25.97 and 33.26% in stressed plants after treating with *P. aeruginosa* (M1) and *B. gladioli* (M2) respectively. Besides, the total carbohydrates were also raised by 23.26 and 35.21% in infected plants in the presence of *P. aeruginosa* (M1) and *B. gladioli* (M2) respectively. The reducing sugar content was also improved by 27.7% after the amendment with *P. aeruginosa* (M1) and 40.87% with *B. gladioli* (M2) treatment (Figure 5).

#### 3.8.2. Effect of Biocontrol Agents on Trehalose, Glycine Betaine, Proline and Free Amino Acid in PPN Infected *L. esculentum* Plants

The PPN infection in *L. esculentum* plants also escalated trehalose, glycine betaine and proline content by 12.06, 16.23 and 34.85% respectively, in comparison to experimental controls. The free amino acid content however, decreased by 30.61% in PPN infected plants when compared to controls. The stimulation in trehalose, glycine betaine and proline levels was seen, subsequently after addition of *P. aeruginosa* (M1) by 61.64, 30.61 and 16.44% respectively. The improvement in trehalose, glycine betaine and proline levels were also recorded in the plants inoculated with *B. gladioli* (M2) by 42.18, 55.93 and 35.71% respectively. Elevation in the free amino acids by 69.12 and 78.04% was noticed in PPN infested plants when inoculating agents *P. aeruginosa* (M1) and *B. gladioli* (M2) were added (Figure 5).

#### 3.8.3. Effect of biocontrol Agents on Organic Acid Profiling in PPN Infected *L. esculentum* Plants

Organic acid profiling was done to assess the levels of fumaric acid, succinic acid, citric acid and malic acid in plants exposed to nematode infestation. An upregulation in the levels of organic acids, fumaric acid (9.7%), succinic acid (29.67%), citric acid (42.26%) and malic acid (57.52%) was noticed in PPN exposed plants in contrast to experimental controls. The drastic elevation in fumaric, succinic, citric and malic acid was recorded by 27.07, 12.02, 43.9 and 118.8% respectively, after the PPN infected plants were amended with *P. aeruginosa* (M1). Similarly, organic acid levels of infected plants inoculated with *B. gladioli* (M2) were also enhanced by 38.05% (fumaric acid), by 7.16% (succinic acid), by 62.66% (citric acid) and by 140.56% (malic acid) respectively (Table 4).

## 4. Discussion

Microbial community present in the rhizosphere have been explored to be useful as natural biocontrol agents in managing PPN infection in *L. esculentum* and improving their growth conditions under normal as well as infectious stages [32]. Our studies indicated that PPN infection declined the growth of 45-days old infected plants in terms of root length, shoot length, plant biomass (fresh and dry weight) and induced gall formation in plants. Earlier also, reduction in the growth attributes have been reported by Gupta et al. [62], *Bacopa monnieri* plants raised under the influence of nematodes. Moreover, reduction in growth of *Salvado rapersica* L. and *S. oleoides* Decne was also reported in terms of decreased plant height, root length, root and shoot biomass, due to PPN infection in the plant species [28]. The reduction in the growth parameters of plants occurs due to infestation of PPN into the roots followed by their further proliferation into interior region leading to development of galls over the root surfaces [63]. Furthermore, the development of galls in root zone of infected plants have also been recorded in the present study. Similar to our observation, the formation of galls have also been noticed in tomatoes and carrots due to the infection of *M. incognita* [19]. They illustrated that nematodes are quite effective in penetrating the root system both fibrous and tap root through secretion of array of compounds to dissolute cell wall for invasion [19]. The present study also depicted that inoculation of *P. aeruginosa*(M1) and *B. gladioli* (M2) improved the morphological characters of the plants inhabited with PPNs. Our studies are in the agreement to previous studies conducted by Abd-El-Khair et al. [64], who reported that biocontrol agents *Bacillus subtilis, B. pumilus* and *P. fluorescens* had a protective role against PPN *M. incognita* in improving root length, shoot length, fresh and dry weights in cowpea. According to their assumptions, the biocontrol agents have the stimulating effects on plants by inducing growth, plant nutrition, producing plant hormones and suppressing various pathogens in the surrounding zones [64]. Moreover, they can also survive under severe conditions to act aggressively against PPNs by producing growth promoting materials in order to cease the negative effects of pathogens [65]. The reduction in the gall number, egg masses and gall index have also been reported by Viljoen et al. [19] in tomato and carrot plants infected with PPNs on supplementation of *B. firmus, B. aryabhattai, B. cereus, Paenibacillus baricinonensis* and *P. alvei* respectively. They concluded that the reduction in the gall formation in the plant species is mainly because of antagonistic effects of these microbial strains on PPNs each possessing its own mode of action to inhibit their proliferation around the root zones [19]. It was also reported that *Bacillus* and *Paenibacillus* sp. are involved in the production of different nematicidal substances that directly inhibit the growth activities of PPNs [66]. Similarly, plant growth promotion has also been studied in nematode infected cucumber plants after the supplementation of *Photorhabdus luminescens* in terms of leaf number, root and shoot length, plant biomass, and fruit yield [67]. The improvement in the growth parameters and reduction in the gall formation of plants in current study might be due to production of extracellular enzymes (serine alkaline protease, chitinases, glucanases etc.), antibiotics and toxins to induce mortality of nematodes [19,32,67]. The main mechanism associated with escalated resistance is the colonisation of the plant species to mediate systemic resistance against parasitic nematodes. This tends to barricade the biting of nematodes to plant roots thereby, inhibiting their penetration inside roots to the maximum extent.

The results of the present study also demonstrated the reduction in the plant pigments in plants infected with nematodes. Our results concur with many similar findings, as it is extensively reported that *Meloidogyne* sp. reduced chlorophyll pigment in tomato plants [68]. They found that disruption in the plant pigments occur mainly due to hindrance in the reactions associated with photosynthesis due to nematode infection, that caused the loss of plant pigments derived during photosynthetic processes [68]. Moreover, nematode damage the root and leaf tissues, chloroplasts and disturb the translocation of photosynthates in plants [69]. The studies conducted in *Capsicum annuum* L. showed a significant reduction in the total carotenoid levels after the inoculation of *M. incognita* [70]. Our results also demonstrated an elevation in the levels of plant pigments in nematode infected *L. esculentum* plants after the rhizobacterial treatment. Our findings coincide with the studies reported by Abd-El-Khair et al. [64], who found the raised levels of *chl*a, *chl*b, total chlorophyll and carotenoid content in nematode infested cowpea plants. Furthermore, a study reported in nematode infected *Pinus pinaster* revealed a decreased incidence of nematode proliferation after microbial treatment along with the prevention in the degradation of plant photosynthetic pigments. They found a significant stimulation in the pigment content in infected plants due to protection of key enzymes involved in photosynthesis from nematode attack [71]. The improved plant pigment levels in the present study are most likely due to protection of photosynthetic apparatus by microbes along with their role in improving translocation of nutrients in plants that ultimately regulates the effective photosynthesis. Moreover, the mechanism entails microbe mediated limitation of PPN migration inside the vascular tissues that ramifies in effective transport of essential minerals required for photosynthesis.

A down regulation in the activities of gas exchange parameters have also been noted in nematode infested plants in the present work. Our studies corroborate with the findings of Goulart, et al. [70], who suggested that nematode infected coffee plants showed remarkable reduction in the transpiration rate, stomatal conductance and CO_2_ concentration. Moreover, they found that overall rate of photosynthesis was lowered in infected plants with reduced starch synthesis [72]. Similarly, the reduction in gaseous exchange parameters, mainly transpiration and assimilation rate in nematode infected *Solanum tuberosum* plants was also recorded [73]. The decrease in the gaseous exchange mechanisms in plants under nematode infection are mainly due to the impairment in the water transport pathways because of root damage by nematodes [74]. Moreover, the reduction in the net photosynthesis accompanied by stomatal regulation and photochemical efficiency was observed in *C. annuum* under the infection of root-feeding parasitic nematodes [70]. The increase in the gaseous exchange parameters was noticed in the present study when an inoculation of *P. aeruginosa* and *B. gladioli* was done. The results of present study coincide with the earlier studies conducted by Mioranza et al. [75] in tomato plants infested by *M. incognita* that showed an elevation in photosynthetic rate, stomatal conductance, internal CO_2_ concentration, leaf temperature and transpiration upon supplementation of *Thuya occidentalis* to infected plants. Furthermore, it was revealed that *M. incognita* infected tomato plants led to enhanced effects upon stomatal conductance and transpiration rate in the presence of *B. lichenoformis* [76]. The acceleration of gaseous exchange parameters in the current study is most likely due to higher synthesis of enzymes involved in photosynthetic processes.

The current study also reported that nematode infection uplifted the levels of oxidative stress markers in plants. Our studies find support from our earlier studies, Khanna et al. [32], where levels of superoxide anions, H_2_O_2_ and MDA were found raised in 10-days old seedlings of tomato under the influence of PPNs. This is directly linked to oxidative damage in plants due to pathogenicity of parasitic nematodes in the form of ROS (reactive oxygen species) generation. Moreover, studies have been reported where levels of total reactive oxygen species, superoxide anions, H_2_O_2_ and PCD (programmed cell death) were elevated in *B. monnieri* L. infected with PPNs [77]. The accumulation of oxidative stress markers and free radicals occurs as a result of toxic by-products of metabolic pathways in plants, when their levels exceed the optimum limits on exposure to parasitic nematodes [78]. Our studies also showed a significant reduction in the levels of all the stress markers in the presence of biocontrol agents. Similar to our studies, the pathogen induced overproduction of ROS has been reported to damage lipid synthesis and biomolecules, that has been considerably alleviated in the presence of microbial agents, implying their role as true antagonists against PPNs [79]. However, the reduction in the levels of MDA have also been observed in *Cicerarietinum* plants, prominently after their treatment with microbial consortium [80]. Another study revealed that nematode infested *B. monnieri* L. plants when inoculated with biocontrol agents (*Chitinphilus* sp. and *Streptomyces* sp.) lowered lipid peroxidation, superoxide anions and hydrogen peroxide levels [77]. They speculated that these inoculating agents had a stimulatory effect in defense mechanisms through reducing the free radicals generated and strengthening their immune system. In addition, they also strengthen the cell membrane of plant tissues that prevent their negative effects on biomolecules and also in preventing their movement towards other parts of the plant [81]. The mechanism associated with the reduced levels of oxidative stress markers in the present study by the aid of microbes is most likely due to elevation in the chain of resistance processes and antioxidative defense genes in plants along with their abilities to directly scavenge the ROS initiated during stress. Along with this, they also restricts their invasion inside the plants so as to balance the redox homeostasis with the plant inner zone.

The up regulation in the activities of enzymatic as well as non-enzymatic antioxidants have also been observed in the present study in plants associated with nematode infection. A study reported in barley infected with PPNs showed enhanced activities of SOD, POD, CAT and PPO [82]. They reported that increase in antioxidative enzymes are relative to resistance mechanisms developed by plants. Moreover, enhanced activities of peroxidases (APOX, GPOX and POD) are directly linked to their involvement in providing structural rigidity to plant tissues through enhanced lignin synthesis in cell walls in order to prevent the nematode penetration in plants [83]. Increases in the activities of peroxidases have also been reported in *Ipomoea batatas* subjected to PPN infection as assessed through transcriptomic analysis [84]. Moreover, an elevation in GPOX, CAT and SOD activities was observed in tomato plants infected by *M. incognita* [85]. The increase in the activities of GR, GST and other antioxidants in plants exposed to nematode infection are due to higher glutathione pools that further activates the enzymes DHAR and GPOX, key enzymes of GR cycle. Also, the level of non-enzymatic antioxidants glutathione, ascorbic acid and tocopherol were enhanced in nematode affected plants in the present study. AsA-GSH pathway is the most important pathway that gets activated during nematode infection in plants. It has been reported to activate the antioxidants as a defense strategy in plants followed by maintaining the redox state during plant-nematode interactions [86]. These antioxidants have been known to directly participate in free radical scavenging, against ROS accumulated during nematode attack [86]. Additionally, tocopherol have been seen to scavenge lipid peroxy radicals accumulated within cell membrane via free radical reactions [87]. Accumulation of different antioxidants have been reported in grape vine by Kesba and El-Beltagi [88] during nematode infection. Our results also showed further upliftment of enzymatic and non-enzymatic antioxidants following treatment with rhizobacterial strains. Our findings corroborate with the studies conducted by Molinari and Leonetti [89], who reported modulation in the activities of defense genes encoding antioxidative system *CAT, APOX* in nematode infected tomato plants. They also reported that SAR (Systemic Acquired Resistance) is the proposed mechanism that activates molecular signaling at different stages of nematode infection within plants, thereby preventing their penetration and movement inside roots [89]. Furthermore, another study reported in tomato plants infected with PPNs showed higher activities of SOD and POD after the inoculation of *Drechslerella dactyloides* and *Dactylaria brochopaga* [90]. According to their research, the microbes participate in the defense signaling during the presence of pathogens, that tend to up regulate the activities of specific biomolecules and antioxidants [90]. Moreover, SOD being a first line of defense in dismutating H_2_O_2_ into non-toxic forms is foremost activated by microbes against ROS generated in cytosol during respiration and photosynthesis [91]. Elevation in the SOD activity and ascorbic acid content have also been reported in nematode infested *B. monnieri* L. plants under the influence of *Chitophilus* sp. and *Streptomyces* sp. [62,77]. Also, the synthesis of non-enzymatic antioxidants were enhanced in these plants. The mechanism associated with stimulatory effects of microbes on antioxidative defense system is most probably due to up-regulation of genes and protein transcripts of antioxidant molecules. And this further attributes to the adaptive responses of plants with protection from PPN infection. The microbes have the capacity to incline the immune responses in plants against toxic environment created within them by advent of PPN proliferation.

Present results also demonstrated the accumulation of phenolic compounds in nematode infested plants. The increased levels of total phenolics have also been reported by Gálvez et al. [70] in *C. annuum* after PPN inoculation in order to induce the resistance in plants. Furthermore, a study conducted in *Oryza sativa* associated with PPN showed higher levels of total phenols (TP) in plants. They depicted that TP induced lignification in epidermal regions of plants, as a constitutive and inducible post penetration mechanism for providing resistance against nematode infection [92]. Flavonoids also act as signaling molecules during plant defense towards PPNs in rhizosphere in modulating auxin transport during gall formation. A study depicting role in flavonoids in nematode parasitized *Medicago truncatula* was conducted, that revealed an induction in the flavonoid pathways to accumulate different flavonoids such as chalcones and flavonones (kaempferol, flavone, isoflavonoids, medicarpin, pterocarpans etc.) to protect them against PPNs and inhibiting gall development in plants [93]. An increase in the levels of anthocyanins was also observed by Labudda et al. [94], in *Arabidopsis thaliana* infected with nematodes as an important antioxidant in protecting the plants from infection. Our study showed a further enhancement in the levels of phenolic compounds when rhizobacterial inoculations were done in PPN infected plants. It was revealed by Nunes da Silva et al. [71] in nematode stressed *P. pinaster* that biosynthesis of phenolics was dramatically increased with the reduction of nematode population at that specific site, being a crucial metabolite in controlling the pathogen prevalence. This is mainly attributed to activation of shikimate biosynthetic pathway by microbes to overproduce metabolites involved in the resistance mechanisms [71]. It was further suggested that inoculation of *D. dactyloides* and *D. brochopaga* in nematode infected tomato plants led to accumulation of phenolic compounds and enzymes involved in its synthesis [90]. They reported that PGPR activated PAL (Phenylalanine ammonia lyase) pathway in roots to improve plant growth, trap nematode population and initiate ISR in plants. Increase in the total phenolic content was also recorded in nematode infected cowpea plants under the influence of different rhizobacterial strains such as *B. subtilis, P. fluorescens* and *B. pumilus* respectively [64]. This phenomenon is quintessential to eradicate the impact of infection from the plants. It can be relatable to direct involvement of phenolic compounds in preventing the impregnation of nematodes inside plant tissues. The phenol deposition at cell walls makes intricate for PPN to biting and invading further inside plants.

Secondary metabolites also known as defense-related metabolites are activated in plants against pathogen attack. They play an important role in defense by triggering direct toxic effects on pathogen or by acting as signaling molecule to activate defense mechanisms in plants [95]. Present study showed a stimulation in the secondary metabolites (osmoprotectants and organic acids) in nematode infested plants. A remarkable increase in the total carbohydrates was observed in *A. thaliana* after the inoculation of PPN [94]. They speculated that an enhancement in the carbohydrates is due to alteration in the photosynthetic efficacy, in order to maintain the permanent inflow of sugars for nematodes prevailing in roots. The amino acid levels were also reported to be lessened in *C. annuum* plants infected with PPNs [70]. They formulated that Leu, Gly, Phe, Cys, Gly, Thr, Val and Ala were found to be declined in infected plants due to the alterations in the expression levels of different genes involved in amino acid metabolism [70]. Moreover, a study conducted in *A. thaliana* roots also showed a higher accumulation of different sugars such as raffinoses, trehaloses, ketoses and kestoses and amino acids (methionine, glutamine, aspartic acid, glutamic acid) in nematode infected plants [96]. According to their analysis, the higher levels of these metabolites produced under nematode infection are mainly due to the fact that they interfere with nematode-plant signaling by reducing the incidence of nematode associations with plants [96]. An elevation in organic acid levels has also been observed in nematode infested *C. annuum* plants [97]. The higher levels of organic acid synthesis in plants have been linked to disease resistance against different pathogens [98]. Our results also demonstrated a further rise in the secondary metabolic profiles in nematode infested plants after amendment with PGPR. Studies have been reported that showed a modulation in the genes involved in amino acid metabolism in nematode infested *A. thaliana* plants inoculated with *Serratia plymuthica* M24T3 [99]. Furthermore, stimulation in the activities of secondary metabolic profiles have also been reported by Oh et al. [100] by *Enterobacter arsburiae* HK169 in nematode infected tomato plants in order to encounter the oxidative stress generated by pathogen attack. However, an upliftment in the secondary metabolic profiles have also been found in tomatoes and carrots exposed to nematode infection after the inoculation of PGPR, *B. firmus* T11, *B. aryabhattai* AO8, *B. cereus*, *P. alvei* T30 and *P. barcinonensis* A10 respectively [19]. The accumulation of organic acids has also been reported by inoculation of *Aspergillus niger* and *A. candidus* in plants exposed to nematode infection to form an antagonistic associations with them [101]. The metabolomic study showed exaggerated levels of secondary metabolite profiles in plants with respect to infection alone as well as with microbes. The main mechanism behind these triggered levels are attributed to activation of genes encoding enzymes involved in synthesis of different secondary metabolites.

## 5. Conclusions

Present work suggested the synergistic role of PGPR in providing rhizosphere community resilience under PPN stress. The association of microbes with plants in promoting growth, metabolic activities and upregulation of defense system to improve plant health and management of PPNs infestation has been explored. The use of microbes as biocontrol agents is the most recent and environment friendly strategy, as they improved the morphological, biochemical, physiological, and secondary metabolic profiles within plants. Moreover, they have also been known to induce ISR in plants along with the production of different toxins, antibiotics, enzymes etc. to control proliferation and penetration of nematodes within plants. They also alter the root exudations that further hinder the mobility of PPN into the soil so that they could not enter into the plant system. Consequently, it has been the most innovative approach to develop strategies for fabrication of most recent technologies in agricultural productivity in ecological manner. Nevertheless, further analysis is essential for better understanding of the mechanisms associated with plant-microbes-PPNs interactions affecting rhizospheric community, where the functions of rhizodeposition is primarily important.

## Figures and Tables

**Table 1:** Effect of M1 (10^9^ cells/mL) and M2 (10^9^ cells/mL) on: ^a^ root length, ^b^ shoot length, ^c^ fresh weight, ^d^ dry weight, ^e^ number of galls in 45-days old *Lycopersicon esculentum* plants under plant parasitic nematodes (PPN) infection.

Treatments	Root Length (cm) (Mean ± SD)	Shoot Length (cm) (Mean ± SD)	Fresh Weight (g/plant) (Mean ± SD)	Dry Weight (g/plant) (Mean ± SD)	No. of Galls (Mean ± SD)
CN	15.78 ± 0.2505 ^b^	22.13 ± 0.4635 ^d^	4.69 ± 0.2495 ^a^	1.96 ± 0.1334 ^b,c^	0
N	8.96 ± 0.6502 ^d^	17.36 ± 0.6036 ^e^	3.04 ± 0.2502 ^c^	1.02 ± 0.1251 ^d^	27.33 ± 1.527 ^a^
M1	17.42 ± 0.3651 ^b^	24.54 ± 0.8015 ^c^	4.71 ± 0.1182 ^a^	1.93 ± 0.0695 ^b,c^	0
M1 + N	15.53 ± 0.7946 ^b,c^	26.80 ± 1.1654 ^b^	4.05 ± 0.2501 ^b^	2.10 ± 0.1100 ^a,b^	19.66 ± 0.1.527 ^b^
M2	19.50 ± 0.7379 ^a^	22.38 ± 0.4178 ^c,d^	4.21 ± 0.1608 ^a,b^	2.02 ± 0.1481 ^b^	0
M2 + N	13.59 ± 1.1119 ^c^	29.47 ± 1.0726 ^a^	4.55 ± 0.1574 ^a^	2.15 ± 0.099 ^a^	22 ± 1 ^b^
F-ratio_(df 1,12)_ T	210.9 **	15.85 **	45.38 **	15.39 **	2520.5 **
F-ratio_(df 2,12)_ D	68.13 **	110.6 **	12.27 **	46.41 **	27.54 **
F-ratio_(df 2,12)_ T × D	20.65 *	80.30 **	31.75 **	43.17 **	24.52 **
HSD	1.949	2.217	0.593	0.321	2.665

Data is presented as means of three replicates ± SD (standard deviation) and HSD values. F ratio values, * indicates significance at *p* ≤ 0.05 and ** indicates significance at *p* ≤ 0.01). Different letters on the graphs indicate that mean values of treatments are significantly different at *p* < 0.5 according to Tukey’s multiple comparison test. CN: Control, N: Nematode, M1: *Pseudomonas aeruginosa*, M2: *Burkholderia gladioli.*

**Table 2:** Effect of M1 (10^9^ cells/mL) and M2 (10^9^ cells/mL) on gas exchange parameters ^a^ net photosynthetic rate, ^b^ stomatal conductance, ^c^ intracellular CO_2_, ^d^ transpiration rate in 45-days old *L. esculentum* under PPN infection.

Treatments	Net Photosynthetic Rate (µmol m^2^ s^−1^) (Mean ± SD)	Stomatal Conductance (mmol CO_2_ m^2^ s^−1^) (Mean ± SD)	Intracellular CO_2_ Rate (µmol mol^1^) (Mean ± SD)	Transpiration Rate (mmol m^2^ s^−1^) (Mean ± SD)
CN	25.54 ± 0.9492 ^c^	0.474 ± 0.016 ^d^	436 ±8.426 ^b,c^	2.453 ± 0.115 ^b^
N	16.87 ± 0.3952 ^e^	0.324 ± 0.0075 ^e^	386.3 ± 6.760 ^d^	1.463 ± 0.1150 ^d^
M1	28.33 ± 0.8304 ^b^	0.524 ± 0.0125 ^c^	455.6 ± 5.511 ^b^	2.75 ± 0.1178 ^a,b^
M1+N	22.44 ± 0.6165 ^d^	0.584 ± 0.0117 ^b^	419.7 ± 8.891 ^c^	1.903 ± 0.1078 ^c^
M2	27.73 ± 0.535 ^b,c^	0.496 ± 0.0091 ^c,d^	442.3 ± 3.146 ^b^	2.97 ± 0.090 ^a^
M2 + N	31.55 ± 1.2690 ^a^	0.611 ± 0.0123 ^a^	480.9 ± 8.603 ^a^	2.13 ± 0.1418 ^c^
F-ratio_(df 1,12)_ T	86.69 **	2.256	21.27 **	266.5 **
F-ratio_(df 2,12)_ D	159.09 **	336.04 **	74.02 **	40.46 **
F-ratio_(df 2,12)_ T × D	96.33 **	205.72 **	65.39 **	1.846
HSD	2.245	0.0326	19.702	0.3171

Data is presented as means of three replicates ± SD and HSD values. F ratio values, * indicates significance at *p* ≤ 0.05 and ** indicates significance at *p* ≤ 0.01). Different letters on the table indicate that mean values of treatments are significantly different at *p* < 0.5 according to Tukey’s multiple comparison.

**Table 3:** Effect of M1 (10^9^ cells/ mL) and M2 (10^9^ cells/ mL) on: SOD, POD, CAT, GPOX, APOX, DHAR, GST, GR, PPO in 45-days old *L. esculentum* plants under PPN infection. Data is presented as means of three replicates ± SD and HSD values. F ratio values, * indicates significance at *p* ≤ 0.05 and ** indicates significance at *p* ≤ 0.01). Different letters on the graphs indicate that mean values of treatments are significantly different at *p* < 0.5 according to Tukey’s multiple comparison test.

Treatments	SOD (Mean ± SD)	POD (Mean ± SD)	CAT (Mean ± SD)	GPOX (Mean ± SD)	APOX (Mean ± SD)	DHAR (Mean ± SD)	GST (Mean ± SD)	GR (Mean ± SD)	PPO (Mean ± SD)
Control	53.44 ± 3.506 ^d^	256.4 ± 6.612 ^f^	54.08 ± 1.859 ^d^	23.58 ± 0.8076 ^e^	389.1 ± 9.030 ^d^	136.8 ± 1.718 ^d^	21.73 ± 1.672 ^e^	210.5 ± 5.574 ^e^	71.13 ± 2.373 ^f^
N	71.97 ± 1.254 ^b^	402.8 ± 1.859 ^c^	70.94 ± 0.941 ^b^	33.65 ± 1.1611 ^c^	442.3 ± 11.220 ^c^	150.5 ± 6.151 ^c^	27.45 ± 1.831 ^c^	353.9 ± 3.156 ^b^	84.82 ± 3.642 ^e^
M1	61.0 ± 1.787 ^c^	349.8 ± 6.733 ^e^	58.77 ± 3.792 ^c,d^	27.35 ± 0.8783 ^d^	651.0 ± 16.274 ^a^	167.1 ± 3.257 ^b^	32.03 ± 2.427 ^c^	286.7 ± 9.087 ^c^	96.28 ± 3.006 ^d^
M1 + N	84.45 ± 2.184 ^a^	524.2 ± 1.539 ^a^	78.62 ± 2.826 ^a^	36.0 ± 0.7885 ^b^	510.9 ± 3.854 ^b^	174.7 ± 2.368 ^a,b^	38.04 ± 1.908 ^b^	360.1 ± 4.650 ^a,b^	137.7 ± 4.165 ^a^
M2	59.33 ± 3.729 ^c,d^	370.3 ± 1.270 ^d^	63.14 ± 2.310 ^c^	25.94 ± 0.3994 ^d^	401.9 ± 2.382 ^d^	156.7 ± 3.503 ^c^	31.41 ± 1.5008 ^c,d^	246.8 ± 13.211 ^d^	111.8 ± 3.856 ^c^
M2 + N	78.74 ± 1.727 ^ab^	454.6 ± 6.143 ^b^	76.50 ± 1.901 ^a,b^	40.1 ± 0.9736 ^a^	522.4 ± 8.446 ^b^	183.2 ± 2.587 ^a^	44.44 ± 2.130 ^a^	379.8 ± 3.659 ^a^	123.8 ± 5.584 ^b^
F-ratio_(df 1,12)_ T	291.8 **	3667.7 **	193.4 **	720.3 **	3.199	90.11 **	81.78 **	11730.7 **	148.3 **
F-ratio_(df 2,12)_ D	23.85 **	849.5 **	18.97 **	40.77 **	246.7 **	113.3 4**	78.78 **	54.79 **	204.4 **
F-ratio_(df 2,12)_ T × D	1.599	142.6 **	4.795 **	16.29 **	155.47 **	11.09 **	6.881 *	37.20 **	26.91 **
HSD	6.967	12.972	6.686	2.375	36.445	9.758	5.308	19.95	10.69

**Table 4:** Effect of M1 (10^9^ cells/mL) and M2 (10^9^ cells/mL) and their combinations on organic acid contents (Fumaric acid, Citric acid, Succinic acid, Malic acid) in 45-days old *L. esculentum* plants under PPN infection.

Treatments	Fumaric Acid (mg g^−1^ FW) (Mean ± SD)	Citric Acid (mg g^−1^ FW) (Mean ± SD)	Succinic Acid (mg g^−1^ FW) (Mean ± SD)	Malic Acid (mg g^−1^ FW) (Mean ± SD)
CN	0.515 ± 0.0060 ^e^	4.337 ± 0.1838 ^e^	0.930 ± 0.0225 ^c^	3.409 ± 0.1758 ^f^
N	0.565 ± 0.0111 ^d^	6.162 ± 0.1476 ^cd^	1.206 ± 0.0338 ^b^	5.379 ± 0.2266 ^e^
M1	0.649 ± 0.0225 ^c^	5.674 ± 0.1402 ^d^	0.985 ± 0.0121 ^c^	6.403 ± 0.1581 ^d^
M1 + N	0.718 ± 0.0193 ^b^	8.878 ± 0.2081 ^b^	1.351 ± 0.0327 ^a^	11.778 ± 0.4950 ^b^
M2	0.611 ± 0.0106 ^c^	6.544 ± 0.1093 ^c^	0.953 ± 0.0106 ^c^	9.237 ± 0.1091 ^c^
M2 + N	0.780 ± 0.0198 ^a^	10.020 ± 0.3984 ^a^	1.413 ± 0.0353 ^a^	12.94 ± 0.3555 ^a^
F-ratio_(df 1,12)_ T	160.7 **	750.6 **	859.22 **	746.3 **
F-ratio_(df 2,12)_ D	173.2 **	297.2 **	33.144 **	866.5 **
F-ratio_(df 2,12)_ T × D	23.83 **	24.24 **	17.907 **	53.17 **
HSD	0.0440	0.6022	0.0729	0.7842

Data is presented as means of three replicates ± SD and HSD values. F ratio values, * indicates significance at *p* ≤ 0.05 and ** indicates significance at *p* ≤ 0.01). Different letters on the table indicate that mean values of treatments are significantly different at *p* < 0.5 according to Tukey’s multiple comparison test.
